# Aneurysm Infection Caused by *Desulfovibrio desulfuricans*

**DOI:** 10.3201/eid2908.230403

**Published:** 2023-08

**Authors:** Tatsuya Fujihara, Keigo Kimura, Hiroo Matsuo, Ryuichi Minoda Sada, Shigeto Hamaguchi, Go Yamamoto, Takuya Yamakura, Satoshi Kutsuna

**Affiliations:** Osaka University Hospital, Osaka, Japan (T. Fujihara, K. Kimura, H. Matsuo);; Osaka University Graduate School of Medicine, Osaka (R. Minoda Sada, S. Hamaguchi, G. Yamamoto, T. Yamakura, S. Kutsuna)

**Keywords:** *Desulfovibrio desulfuricans*, aneurysm, infected, RNA, ribosomal, 16S, bacteria, bacterial infection, anaerobic, Japan

## Abstract

An 84-year-old man in Japan who had undergone endovascular aortic repair 9 years earlier had an infected aneurysm develop. We detected *Desulfovibrio desulfuricans* MB at the site. The patient recovered after surgical debridement, artificial vessel replacement, and appropriate antimicrobial therapy. Clinicians should suspect *Desulfovibrio* spp. infection in similar cases.

*Desulfovibrio* species are gram-negative, sulfate-reducing, nonfermenting, anaerobic bacteria found in the environment, including in soil, water, and sewage, as well as in the digestive tracts of humans and animals (*1*). They rarely cause human infections, and their clinical prevalence and features are unclear (*2*,*3*). We describe a case of an infected aneurysm caused by *Desulfovibrio desulfuricans* MB that was treated successfully with artificial vessel replacement and antimicrobial therapy after identification of the causative pathogen.

An 84-year-old man who had undergone endovascular aortic repair (EVAR) 9 years earlier was referred for suspected mycotic thoracoabdominal aortic aneurysm. His medical history included hypertension, type 2 diabetes mellitus, and chronic kidney disease. Six weeks before referral, he had eaten grilled fish; 2 weeks later, he had experienced fever with transient chills, followed by persistent abdominal pain for 2 weeks. Computed tomography revealed a fishbone lodged in the ileocecal tract, with a hyper-absorptive zone in the arterial wall; the post-EVAR abdominal aortic aneurysm was larger than it had been 4 months before. He had received antimicrobial therapy 5 days before referral. Blood cultures after treatment were negative.

At admission, the patient did not appear distressed. Blood pressure was 112/80 mm Hg, pulse rate 60 beats/min, body temperature 36.4°C, respiratory rate 18 breaths/min, and Glasgow coma scale score 15. On physical examination, chest and cardiovascular findings were unremarkable; abdominal tenderness was noted on palpation. Laboratory tests indicated that leukocyte count was 11.05 × 10^3^ cells/L, C-reactive protein 12.12 mg/dL, serum creatinine 1.39 mg/dL, and hemoglobin A1c level 9.9. Results of additional blood culture performed under treatment with ampicillin/sulbactam (3 g/6 h) was also negative.

On day 5 of admission, surgical debridement was performed, followed by partial removal of the EVAR graft and in situ Y-graft placement with revascularization, including the bilateral renal and superior mesenteric arteries. Intraoperative findings showed partial abscess formation in the abdominal artery wall. Gram stain of the abscess pus showed gram-negative rods (Figure, panel A), and subsequent anaerobic intraoperative pus cultures were observed (Figure, panel B). Matrix-assisted laser desorption/ionization time-of-flight (MALDI-TOF) mass spectrometry using MALDI Biotyper library version 9 (Bruker Daltonics, https://www.bruker.com) did not reliably identify the pathogens (Appendix). We therefore conducted 16S ribosomal RNA (16S rRNA) sequencing of the isolates by using the BLAST sequence homology search program (EzBioCloud, https://www.ezbiocloud.net) for analysis. Sequencing showed 99.86% homology with *D. desulfuricans* MB ATCC27774 (GenBank accession no. CP001358.1) and 1,470/1,472 bp nucleotide matches. The desulfoviridin test was positive, and the biochemical properties of the isolates were consistent with those of *D. desulfuricans* MB. On day 6, we performed thoracic endovascular aortic repair, coil embolization of the celiac and inferior mesenteric artery, and colonoscopic fishbone resection.

**Figure:**
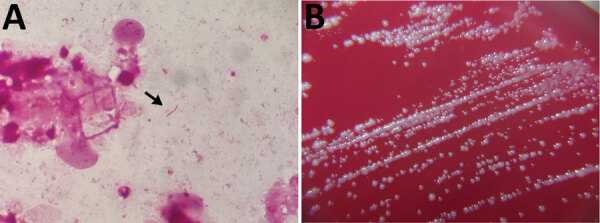
Detection and colonization of *Desulfovibrio desulfuricans* in an 84-year-old man in Japan who had undergone endovascular aortic repair 9 years earlier*.* A) Gram stain of pus. *D. desulfuricans* MB has a gram-negative spiral rod appearance (arrow). Original magnification ×1,000. B) Colonies of *D. desulfuricans* MB on ABHK agar. Biochemical properties showed positive results for catalase and negative for indole and urease. In vitro susceptibility testing revealed that it had the following MICs: meropenem, <2 μg/mL; cefotaxime, <2 μg/mL; ampicillin/sulbactam, <4 μg/mL; piperacillin/tazobactam, <16 μg/mL; and clindamycin, >8 μg/mL.

The postsurgical course and antibiotic treatment were uneventful. The patient received 4 weeks of ampicillin/sulbactam (3 g/6 h) treatment after surgical debridement. He was discharged with continued oral administration of amoxicillin/clavulanate. Because the infected EVAR graft was retained, ongoing antibiotic treatment was recommended.

*D. desulfuricans*, which has 2 genotypes (Essex and MB), is the most pathogenic among *Desulfovibrio* spp., and bacteremia occurs when there is bacterial translocation from the intestinal tract (*3*–*5*). *Desulfovibrio* spp. can also cause appendicitis, abdominal abscesses, and septic arthritis; however, an infected aneurysm is extremely rare, and only 2 cases, including 1 suspected case, have been previously reported (*3*,*6*). One of the reasons for the rarity is that *Desulfovibrio* spp. require a long incubation period (3–7 days) for detection in blood culture, and they cannot be identified by using biochemical reaction tests (*3*,*7*). Except in 2 instances, previous cases have also required identification using 16S rRNA. *Desulfovibrio* spp. were not included in many system databases until recently, possibly contributing to the inability to identify them using MALDI-TOF mass spectrometry (*2*). However, even though *Desulfovibrio* spp. are included in the MALDI-TOF mass spectrometry library we used, we were unable to identify the pathogen. Factors such as the absolute number of strains registered in the *Desulfovibrio* spp. library may make identification difficult, and the accuracy of identification may still be problematic. Difficulty in identification may therefore result in underdiagnosis.

In our patient, the fishbone perforated the ileocecal region, enabling hematogenous bacteremia to enter and cause the infection. Uncontrolled type 2 diabetes may also have played a role. No pathogens were detected in the blood cultures, possibly because of previous administration of antimicrobial therapy. However, after consultation with the infectious disease specialist and microbiologist, we performed 16S rRNA sequencing, which led to detection of *D. desulfuricans* MB and appropriate antimicrobial administration. When gram-negative bacilli are detected in anaerobic cultures of infected aneurysms, *Desulfovibrio* spp. infection should be suspected, especially when gastrointestinal disease is present.

In general, *Desulfovibrio* spp. are susceptible to chloramphenicol and metronidazole; most are susceptible to imipenem and clindamycin, but the optimal treatment for *Desulfovibrio* spp. infection has not been established (*3*,*7*). Our report suggests that ampicillin/sulbactam and amoxicillin/clavulanate may also be effective therapeutic options for *Desulfovibrio* spp. infections.

Appendix Additional information about infected aneurysm caused by *Desulfovibrio desulfuricans* infection.
