# Supplementary material for: Aneurysm Infection Caused by Desulfovibrio desulfuricans
Source: Emerg Infect Dis. 2023 Aug;29(8):1680–1. doi: 10.3201/eid2908.230403 (PMC10370862; doi:10.3201/eid2908.230403)
Supplement: Appendix — Additional information about infected aneurysm caused by Desulfovibrio desulfuricans infection. [file 23-0403-Techapp-s1.pdf]

Article DOI: https://doi.org/10.3201/eid2908.230403

EID cannot ensure accessibility for supplementary materials supplied by authors. Readers who have difficulty accessing supplementary content should contact the authors for assistance.

Infected Aneurysm Caused by *Desulfovibrio desulfuricans* Infection

Appendix

Appendix Table. Identification scores obtained by MALDI-TOF mass spectrometry (3 attempts)*

Rank	Matched pattern	Score
Attempt 1		
Rank	Matched pattern	Score
1	*Enterococcus mundtii* 3783_2016 IMHM	1.34
2	*Paenibacillus macerans* DSM24T DSM	1.31
3	*Campylobacter jejuni* MB_6111_05THL	1.26
4	*Prevotella melaninogenica* IBS _MS_43IBS	1.25
5	*Aerooccus viridans* CC 6210 MCW	1.24
6	*Aerooccus viridans* CC 6213 MCW	1.22
7	*Enterococcus mundtii* CCUG 30574 CCUG	1.21
8	*Lactobacillus perolens* DSM 12745 DCM	1.20
9	*Escherichia coli* B421 UFL	1.18
10	*Paembacillus glucanolyticus* DSM 5162T DSM	1.18
Attempt 2		
Rank	Matched pattern	Score
1	*Campylobacter jejuni* ATCC 29428	1.46
2	*Enterococcus mundtii* 3787_2016 IMHM	1.31
3	*Campylobacter jejuni* MB_4738_05 THL	1.39
4	*Campylobacter jejuni* MB_7240_05 THL	1.34
5	*Paenibacillus lautus* DSM 13411 DSM	1.32
6	*Campylobacter jejuni* MB_6111_05 THL	1.29
7	*Enterococcus faecium* PX_21086109_III MLD	1.27
8	*Enterococcus mundtii* CCUG 30574 CCUG	1.26
9	*Campylobacter upsaliensis* 451_01 NVU	1.25
10	*Enterococcus mundtii* LMG 20698 LMG	1.24
Attempt 3		
Rank	Matched pattern	Score
1	*Enterococcus mundtii* 3783_2016 IMHM	1.42
2	*Campylobacter jejuni* ATCC 29428 THL	1.36
3	*Campylobacter jejuni* MB_7240_05 THL	1.35
4	*Paenibacillus macerans* DSM 24T DSM	1.34
5	*Paenibacillus macerans* IBS_MS_1 IBS	1.32
6	*Enterococcus mundtii* LMG 20698 LMG	1.28
7	*Campylobacter jejuni* MB_4738_05 THL	1.25
8	*Campylobacter jejuni* MB_6111_05 THL	1.25
9	*Acidovorax avenae* ssp *avenae* DSM 7227T HAM	1.21
10	*Lactobacillus murinus* DSM 20453 DSM	1.21

*MALDI-TOF, matrix-assisted laser desorption/ionization time-of-flight mass spectrometry (MALDI Biotyper: Bruker Daltonics, Germany, library version 9). Identification Method: MALDI Biotyper MSP Identification Standard Method 1.1. Preprocessing Method: MALDI Biotyper Preprocessing Standard Method 1.1. ACQ Method: D:\Methods\flexControlMethods\MBT_FC.par. AutoXecute Method: MBT_AutoX_smart. Consistency Category (based on 2 best matches): C (each time). Applied Taxonomy Tree: Bruker Taxonomy.
